# The Sound Games: Introducing Gamification into Stanford’s Orientation on Emergency Ultrasound

**DOI:** 10.7759/cureus.1699

**Published:** 2017-09-18

**Authors:** Viveta Lobo, Andrew Q Stromberg, Peter Rosston

**Affiliations:** 1 Department of Emergency Medicine, Stanford University School of Medicine; 2 Emergency Medicine, Georgetown Medical School; 3 Chemistry, Washington University in St. Louis

**Keywords:** point-of-care ultrasound, emergency ultrasound, simulation, ultrasound orientation

## Abstract

Point-of-care ultrasound is a critical component of graduate medical training in emergency medicine. Innovation in ultrasound teaching methods is greatly needed to keep up with a changing medical landscape. A field-wide trend promoting simulation and technology-enhanced learning is underway in an effort to improve patient care, as well as patient safety. In an effort to both motivate students and increase their skill retention, training methods are shifting towards a friendly competition model and are gaining popularity nationwide. In line with this emerging trend, Stanford incorporated the Sound Games – an educational ultrasound event with a distinctly competitive thread – within its existing two-day point-of-care ultrasound orientation course for emergency medicine interns. In this study, we demonstrate successful implementation of the orientation program, significant learning gains in participants, and overall student satisfaction with the course.

## Introduction

Point-of-care ultrasound (POCUS) has proven to be a valuable tool in patient care within the emergency department. POCUS skills are mandated milestones instituted by the Accreditation Council for Graduate Medical Education (ACGME) for all emergency medicine (EM) residency programs across the United States [1-2]. These skills are taught in a multitude of ways, including didactic lectures and hands-on workshops. Competency in POCUS is tested through both questions and practical ability to perform ultrasound applications. This dual testing method is essential because it assesses the residents' general breadth of knowledge, as well as their aptitude in the acquisition and interpretation of images [3-5]. In 2008, the American College of Emergency Physicians (ACEP) specified in their Emergency Ultrasound (EUS) Guidelines Policy Statement that emergency medicine residents can follow two different paths to competence in emergency ultrasound training: the residency-based pathway or the practice-based pathway. For both tracks, it is recommended that at least a one-day introduction/orientation be given, using a variety of different teaching mediums including videos, lectures, journal review, and hands-on practice. In the Department of Emergency Medicine at Stanford University, all interns participate in a two-day POCUS orientation, in addition to completing a two-week rotation in ultrasound during their first year of residency. Graduate medical education has remained largely unchanged from the historically accepted apprentice-based learning model. This paradigm of “see one, do one, teach one” has been going out of favor as the medical field seeks to provide safer and higher quality care to patients. Evolving this time-honored paradigm of clinical education to reflect modern advances could significantly improve patient safety. Taking inspiration from other high-reliability organizations, such as the commercial airline business, many medical training programs are turning to simulation, which offers hands-on practice in a risk-free environment. The growth of patient simulation as a core skill acquisition tool has been driven by a number of factors: declining inpatient populations, concerns for patient safety, and advances in learning theory. Likewise, the incorporation of academic competitions (gamification) into training programs has demonstrated a track record of success. Gamification is the use of games or game elements in non-game settings, ideally to increase the involvement, focus, learning, or productivity of students. Theories behind the value of gamification in an educational setting have been prevalent for decades; however, gamification has only recently been adopted in many different sectors. At the 2012 Society for Academic Emergency Medicine (SAEM) annual conference, the Academy of Emergency Ultrasound successfully executed an ultrasound competition for emergency medicine residents that they called the SonoGames [6]. The SonoGames is now an annual, four-hour, interactive competition aimed at assessing and improving senior emergency medicine residents’ knowledge of POCUS, imaging ability, and clinical decision making. The number of residents participating in the SonoGames has more than doubled since its inauguration and has continued to be an effective and fun learning experience. Inspired by the SonoGames, Stanford’s Department of Emergency Medicine sought to create an innovative and comprehensive learning tool – the Sound Games – to augment their existing mandatory intern orientation course. To our knowledge, no such educational model has been created for first-year EM residents. The competition was integrated into the already established two-day mandatory POCUS intern orientation and included both question rounds and a hands-on practical round using simulation.

## Materials and methods

Preparation

Emergency ultrasound (EUS) faculty devised a pretest to assess the participants’ current levels of training on point-of-care ultrasound. Pretest questions covered basic technical skills, image interpretation, and patient management - all standard ACEP-specified topics introduced during the mandatory two-day POCUS orientation. In addition, interns were asked to complete a subjective self-assessment of their comfort with POCUS. One week prior to the orientation, participants completed the mailed pretests. The Stanford University Institutional Review Board approved the protocol (#38214).

Participants

All incoming Stanford Emergency Medicine interns participated in this pilot study during their two-day POCUS orientation course. Sixteen residents (nine male and seven female) formed our study cohort. One-third (38%) of interns stated in the pretest that they had minimal experience with ultrasound, defined as being able to turn on the machine and locate the liver. The other 62% stated that they had intermediate experience with ultrasound, defined as being able to perform basic ultrasound applications, such as a focused assessment with sonography for trauma (FAST) exam or an echocardiogram. Four interns had participated in four-year longitudinal curriculums that included POCUS, and another four interns reported taking one or more formal POCUS electives in medical school. The remaining eight interns were classified as having no prior formal ultrasound training. Using the pretests and self-assessments as guides, eight competing teams of two interns each were formed, paired to balance previous knowledge and skill across teams.

Competition structure

**Figure 1:**
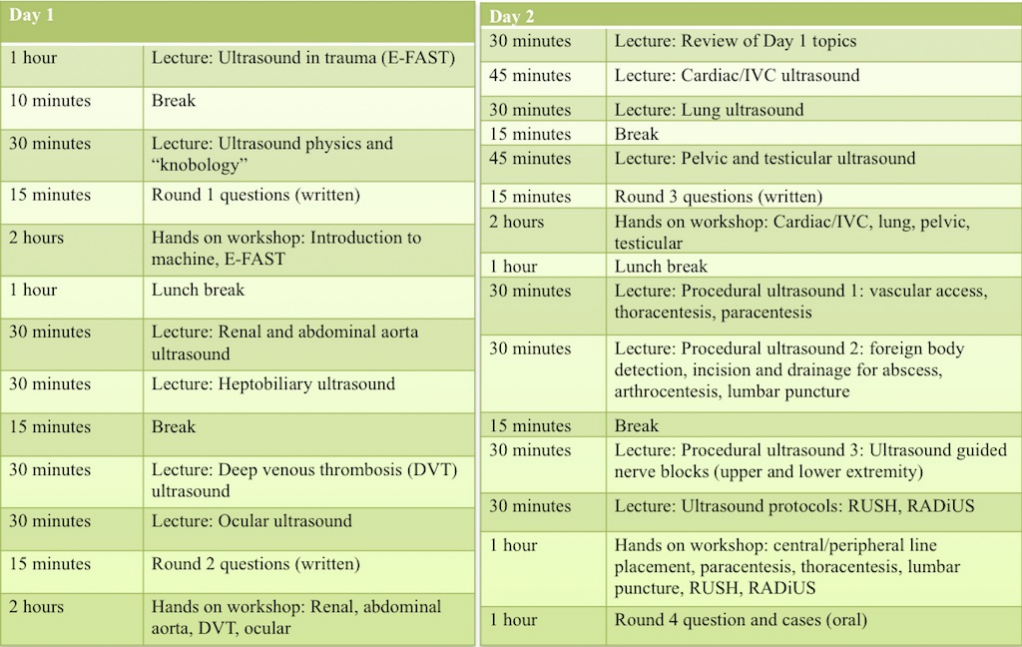
Two-day schedule for intern orientation in ultrasound, including question rounds. E-FAST:  extended focused assessment with sonography for trauma; IVC: inferior vena cava; RADiUS: rapid assessment of dyspnea with ultrasonography; RUSH: rapid ultrasound in shock

The Sound Games' competition was integrated into the two-day POCUS orientation and included three rounds of questioning and one hands-on practical session using simulation (Figure 1). All competition questions and simulation scenarios were written and reviewed by EUS faculty.

Question rounds

**Figure 2:**
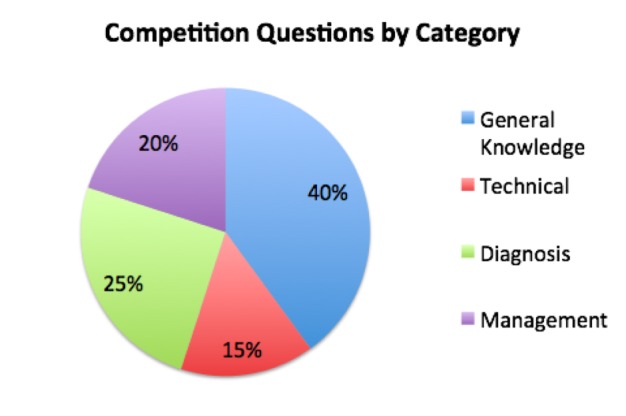
Competition questions by category Sound Games' questions covered material from four distinct categories: general knowledge, technical, diagnostic, and management

**Figure 3:**
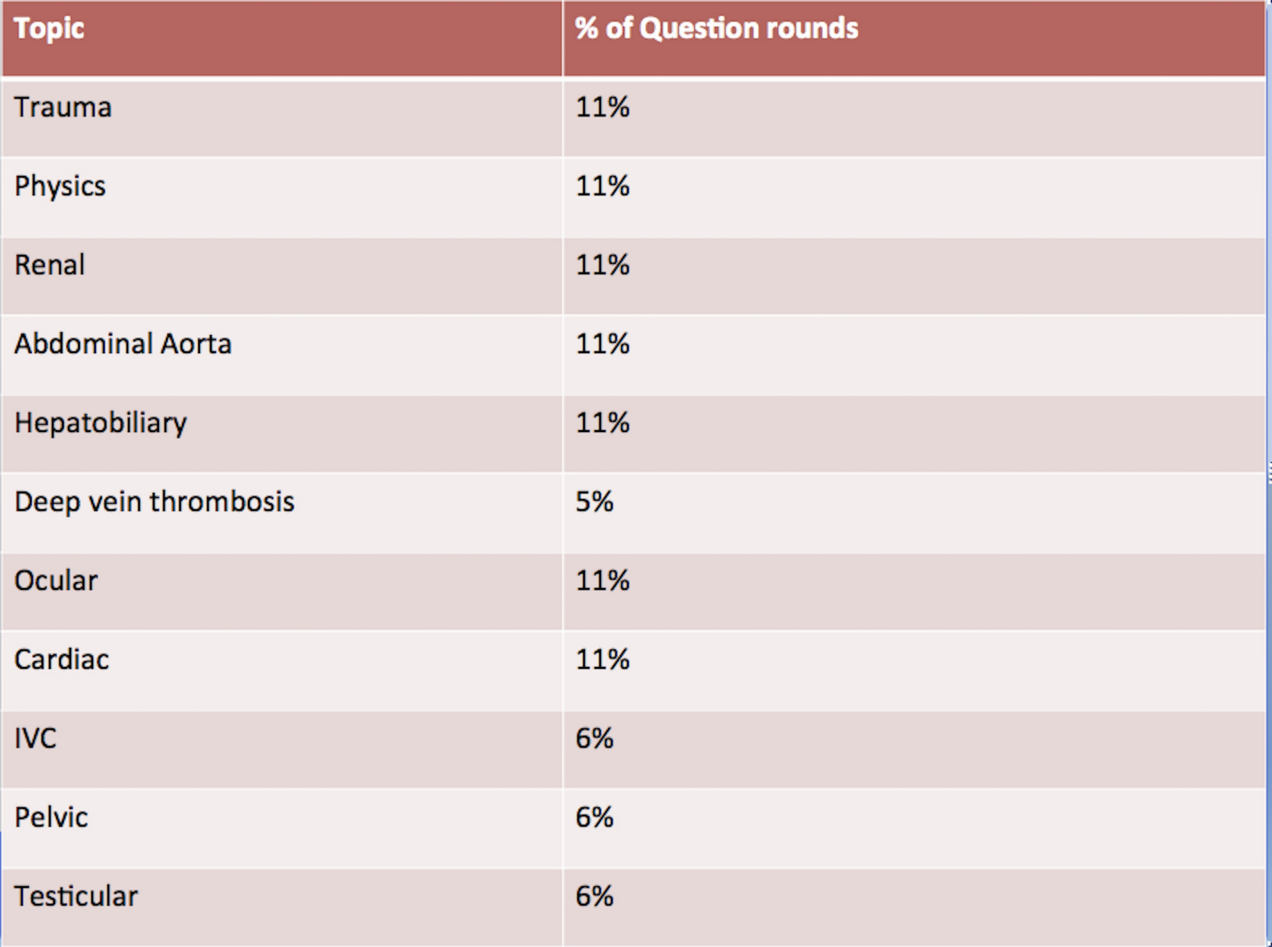
Competition categories by topic The percentage of questions in the three question rounds addressing each topic from the American College of Emergency Physicians-specified curriculum. IVC: inferior vena cava

Intern teams competed in three question rounds throughout the orientation. Question rounds were interspersed between blocks of traditional lectures, with each round testing material presented in the lectures immediately preceding that round. Questions were projected on a screen and simultaneously read aloud by a moderator after which each team was given two minutes to answer the questions in written form. Answer sheets were collected following the completion of each round. Questions were presented in various formats – multiple choice, true or false, open-ended – and spanned four categories: general knowledge, technical, diagnostic, and management (Figure 2). Each question round covered select material from the ACEP-specified curriculum. Round one covered trauma and ultrasound physics. Round two encompassed renal, hepatobiliary, abdominal aorta, deep vein thrombosis, and ocular studies. Round three covered echocardiography, inferior vena cava, lung, obstetrics/gynecology (OBGYN), and testicular ultrasound (Figure 3). Each question round was followed by a traditional hands-on workshop with multiple scanning stations. All questions were equally weighted in the final scoring.

Simulation round

At the end of the second day, all teams competed in a practical simulation round that tested their ability to correctly utilize POCUS in the clinical setting. The integrated technology of Laerdal simulation SimMan (Laerdal Medical Corp., Stavanger, Norway) and the SonoSim LiveScan (Sonosim, Santa Monica, CA) was used during the simulation phase. For the practicum, each team was given a comparable, short, clinical vignette and instructed to present their assessment before the classroom. Teams scored one point if they correctly diagnosed the case based on their POCUS acquisition and interpretation. A second point was given if they correctly identified the next step in clinical management. A total of three minutes was allotted to provide both answers. Response times were recorded for each team. Simulation cases represented one of seven potential diagnoses: cardiac tamponade, tension pneumothorax, ruptured abdominal aortic aneurysm, pneumonia, hemoperitoneum, hemothorax, or unexplained low blood pressure. Judges were two fellowship-trained EUS physicians.

Scoring

Each correct answer during the three question rounds was conferred one point, for a possible 18 total points. Teams could score an additional two points during the simulation round. In the event of a tie, the team with the faster response time in the simulation round would be declared the winner.

Follow-up and analysis

At the end of the orientation course, participants were sent a post-test consisting of the same objective pretest questions with an additional subjective survey. Comparison of performance between the pre and post-test was used to quantify learning. The subjective survey gauged intern satisfaction with the competition, teamwork, perceived knowledge benefit, and value of the Sound Games, as well as a self-assessment of their comfort with POCUS. Participants also submitted feedback on the course as a whole to help guide future directions. The pre and post-tests were not part of the competition but rather a tool for tracking teaching effectiveness and learning. The primary outcome measure for this study was successful learning demonstrated by a statistically significant improvement in mean score between the pretest and post-test. As a secondary outcome measure, researchers looked at participants’ perception of the gamification experience using a Likert scale.

## Results

The Sound Games competition finished with two groups tied for first place, with response times during the simulation round being used as the tiebreaker. The winning team scored 18/20 total points. The range of scores among the remaining seven teams was 14-17 points. One question was removed from scoring for all teams because it was deemed to have been ambiguous. The mean pretest score was 6.9/10, and the mean post-test score was 8.1/10. The measured difference of 1.19 (13%) between pre and post-tests was statistically significant (p < 0.05).

**Figure 4:**
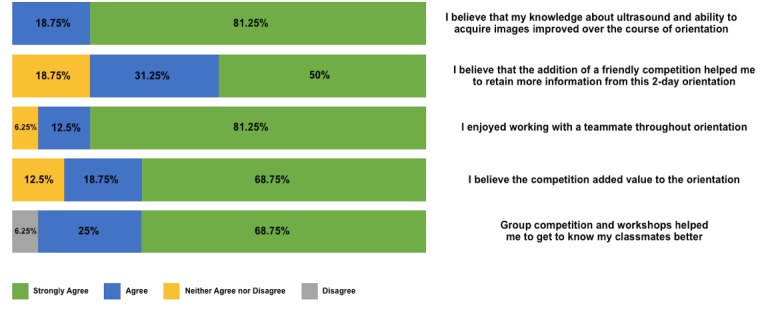
Subjective survey results measured satisfaction with the competition, teamwork, perceived knowledge benefit, and value.

The post-test survey results indicated that 13/16 (81%) "strongly agreed" that working in a team was enjoyable, and that 11/16 (69%) "strongly agreed" that the competition format helped them become better acquainted with their peers. Likewise, 11/16 (69%) "strongly agreed” that the Sound Games component added value to the overall orientation. All participants agreed that their knowledge of ultrasound improved following completion of the two-day training, with 13/16 (81%) stating that they “strongly agreed”. Similarly, 13/16 (81%) agreed that the competition helped them learn the material better (Figure 4).

## Discussion

This study explored the effects of gamification, with the incorporation of simulation, on learning and enjoyment during point-of care-ultrasound training for emergency medicine interns. Our results suggest that the addition of a competitive element (the Sound Games) to our already established mandatory orientation course was both successful from a learning perspective and enjoyable for participants. Interns felt that the additional competition aspect of the orientation added a supplementary educational value. This assertion has been corroborated in the literature. Many studies have assessed the efficacy of gamification as a learning tool, beyond just its basic implementation as a programmatic component. The most comprehensive review, carried out by Hamari, et al., concluded that gamification does increase learning overall (Hamari J, Koivisto J, Sarsa H: Does gamification work? A literature review of empirical studies on gamification. Presented at the 47th Hawaii Internatl. Conf. on System Sciences, Jan. 6-9, 2014; doi: 10.1109/HICSS.2014.377). Other studies went further, noting increases in student participation – both in-class and on assignments – due to the gamification of a course. However, Koivisto and others determined that the educational benefits of gamification differ on a case-by-case basis [7]. We measured a statistically significant improvement in pre and post-test scores among the participants, suggesting that interns were able to synthesize material presented from multiple learning modalities during the orientation. In addition to gamification, this study also utilized simulation technology as a component of the Sound Games. While most ultrasound applications are non-invasive procedures, building experience and confidence through simulation is becoming an increasingly important part of medical teaching. Research has shown that simulation offers a valuable hands-on training opportunity without placing patients at risk. Sites, et al. demonstrated that there is a learning curve for inexperienced anesthesia residents learning to perform ultrasound-guided tasks and that new residents were able to rapidly learn and improve their speed and accuracy in performing a simulated interventional ultrasound procedure [8]. Similarly, Rodriguez-Paz, et al., supported the incorporation of simulation into resident training as a major future direction in patient safety research, debunking the previous paradigm of “see one, do one, teach one” [9]. In fact, a large meta-analysis of trials studying simulation has shown that compared to no intervention, technology-enhanced simulation training was consistently associated with large learning gains and moderate effects for patient safety [10]. Therefore, research suggests that increasing our residents’ exposure to additional simulation and gamification opportunities, such as that offered by the Sound Games, during their early training will be beneficial. Lastly, the survey results suggest that the course was well-received overall, with the majority of participants stating that the competitive format added value to their orientation. The interns also stated that the Sound Games contributed to building class-camaraderie and encouraging improved communication among residents. As efficient emergency department functioning demands effective teamwork and communication to succeed, encouraging these valuable interactions early in residency may be advantageous as the interns progress through their training. Individual feedback responses following course completion were largely positive: “This was a well-done course that greatly increased my skills with ultrasound. I enjoyed the team dynamic and team building functions.” and “The competition was very fun and the collaborative team component was one of the highlights.”.

Limitations

Due to the mandatory nature of Stanford's POCUS orientation and the limited number of entering interns, one major limitation of the study was the inability to have a control group that participated in the orientation but not the competition component. Therefore, we cannot assess what amount, if any, of the learning gains documented between the pre and post-tests is attributable to the Sound Games specifically. While gains cannot be quantified, the survey indicated that the interns believed the Sound Games contributed directly to improvements in their POCUS-specific knowledge and skill. Going forward, the collaboration between two different residency programs – one utilizing a competition model, while the other excludes this component – could clarify the direct effects of gamification on knowledge acquisition. Similarly, the pre and post-tests would benefit from expansion to include hands-on testing to obtain a broader assessment of learning gains.

## Conclusions

The Sound Games effectively demonstrated that gamification and simulation can be incorporated into graduate medical training to both stimulate learning and to improve participant enjoyment. Thus, both teaching mediums offer potential opportunities for future educational innovation in medicine as the field looks for novel ways to train residents. The Sound Games is a valuable training model with the ultimate goal of interactive and engaging education while promoting patient safety and care. 
